# Adherence to stand-by emergency treatment and mosquito protection measures in short-term travellers to moderate malaria risk areas

**DOI:** 10.1016/j.nmni.2024.101561

**Published:** 2025-01-01

**Authors:** Daniel Julien Franken, Vita Willemijn Jongen, Anna Rooyakkers, Martin Peter Grobusch, Jelte Elsinga, Margarita Boering, Maria Prins, Brigitte Antonia Geertruida Lucía van Cleef

**Affiliations:** a Public Health Service of Amsterdam, Department of Infectious Diseases, Nieuwe Achtergracht 100, Amsterdam, the Netherlands; b Amsterdam UMC Location University of Amsterdam, Department of Infectious Diseases, Meibergdreef 9, Amsterdam, the Netherlands; c Dutch Coordination Centre for Travellers' Health Advice (LCR), Nieuwe Achtergracht 100, Amsterdam, the Netherlands; d Stichting Hiv Monitoring, Tafelbergweg 51, Amsterdam, the Netherlands; e Amsterdam UMC Location University of Amsterdam, Institute for Immunology and Infectious Diseases (AI&I), Meibergdreef 9, Amsterdam, the Netherlands; f Amsterdam UMC Location University of Amsterdam, Centre of Tropical and Travel Medicine, Department of Infectious Diseases, Meibergdreef 9, Amsterdam, the Netherlands; g Amsterdam Public Health Research Institute (APH), Meibergdreef 9, Amsterdam, the Netherlands; h Amsterdam UMC Location University of Amsterdam, Department of Medical Microbiology and Infection Prevention, Meibergdreef 9, Amsterdam, the Netherlands

**Keywords:** Malaria, Prevention and control, Mosquito nets, Mosquito control, Mosquito-borne diseases, Travel-related illness

## Abstract

**Background:**

Malaria remains a threat to travellers to (sub)tropical regions. This study assessed adherence to malaria prevention measures among travellers to moderate-risk malaria areas, including the use of standby emergency treatment (SBET), healthcare-seeking behaviour during fever, and mosquito protection measures.

**Methods:**

We analysed data from adult travellers to moderate-risk malaria areas participating in a prospective study (2018–2023) at the Public Health Service of Amsterdam, the Netherlands. Participants maintained a daily diary during travel, recording questions about adherence to mosquito protection measures, symptoms, SBET use, and seeking medical help. In case of fever, participants were instructed to measure their temperature, use SBET if in a remote area, and seek medical help. We used Poisson regression to assess determinants for adherence to mosquito protection measures.

**Results:**

Of 686 recruited travellers, 405 (59 %) completed the diary. Of these travellers 44 % received a pre-travel SBET prescription, although presumably only a small fraction of them actually travelled remotely. None of the 25 travellers who reported fever used the prescribed SBET and five sought medical care. Thirty-five percent of participants used DEET and 5 % used a mosquito net on ≥75 % of the nights with malaria risk. Longer travel duration was associated with lower adherence to DEET use.

**Conclusions:**

Few travellers with fever used SBET or sought medical care, despite their pre-travel advice. To reduce costs and medication spillage, SBET should only be advised to travellers who travel to very remote regions where medical help is inaccessible. Further research should focus on the behavioural concepts underlying these choices.

## 1 Introduction

Malaria is a life-threatening disease caused by *Plasmodium* spp. parasites, and is transmitted through the bite of an infected female anopheline mosquito [1]. In 2022, 249 million cases of malaria were reported globally, with an estimated 608,000 deaths attributed to malaria [2]. Travellers to tropical and subtropical regions are at risk of contracting malaria and potentially becoming seriously ill, with approximately 30,000 travel-related malaria infections occurring annually [[3], [4], [5]]. In 2022, 6,131 confirmed malaria cases were reported in the EU/EEA, almost all travel-related [6]. To protect travellers from malaria, joint expert committees from Belgium, Germany, the Netherlands and Switzerland developed recommendations and malaria maps outlining transmission-risk [7,8]. Malaria transmission-risk has been categorized as low, moderate or high, based on the annual parasite index of *P. falciparum* and *P. vivax* in the local population, access to healthcare and, if available, the incidence of travel-related imported malaria cases [7,9].

Recommendations for malaria prevention depend on malaria risk as well as other travel-related factors such as duration of travel and access to healthcare. Additionally, traveller-related factors are considered, including the travellers' health, age, pregnancy status, medication use, and personal preferences [9]. According to the Dutch Coordination Centre for Travellers’ Health Advice (LCR) guideline, mosquito protection measures form the basis to prevent malaria in all risk areas [7,9]. These measures include using a mosquito repellent containing N,N-diethyl-3-methylbenzamide (DEET), sleeping under an impregnated mosquito bed net, sleeping in an air-conditioned room, and wearing clothes that cover most of the body [9]. Chemoprophylaxis is advised in all high-risk malaria areas, which mainly includes countries in Sub-Saharan Africa [9].

The advice given to travellers to moderate-risk regions, primarily in South America and Southeast Asia, is less straightforward, leading to different national prevention recommendations [8,10]. Switzerland was the first country to start recommending standby emergency treatment (SBET) to travellers to moderate-risk areas, followed by other European countries including the Netherlands [[9], [10], [11], [12]]. SBET is intended for use in case of fever when malaria is suspected, and prompt medical assistance is unavailable [9,12]. However, two systematic reviews showed that travellers had difficulty to follow these recommendations [13,14]. Furthermore, medical infrastructure has greatly improved over the last decades with adequate diagnostics and treatment available in many malaria-endemic countries [3]. Therefore, in September 2021, the national LCR guideline was revised and recommend to only prescribe SBET to travellers who are unable to seek medical care within 48 hours.

At present, research on healthcare-seeking behaviour of travellers with fever, the utilization of SBET and the underlying factors influencing travellers’ decision during fever is limited [3,15]. Therefore, the aim of this study was to (i) assess adherence to advice for febrile travellers (use of SBET and consulting a medical doctor); (ii) assess adherence to mosquito protection measures of travellers to moderate malaria risk areas, and (iii) explore socio-demographic and travel-related determinants of non-adherence to mosquito protection measures. The results of this study can help optimize malaria-preventive recommendations for travellers to moderate malaria risk areas.

## 2 Materials and methods

### 2.1 Study setting

The study was conducted between October 15, 2018; and September 21, 2023, at the Travel Clinic of the Public Health Service in Amsterdam, the Netherlands. This travel clinic sees approximately 25,000 travellers each year, of whom approximately 2,000 travel to moderate malaria risk areas. Travellers have to pay for the pre-travel consultation, vaccinations, and malaria prescription. Travellers are advised (during the consultation and in writing) to use mosquito protection measures (i.e., using mosquito repellent during the evening/night, sleeping under an impregnated bed net, using an air-conditioning during the night and wearing long clothing) and are advised to seek medical help when experiencing fever (>38.5 °C or feeling feverish) seven days or more after arrival in malaria area in accordance with the national LCR-guidelines [9]. Socio-demographic and travel-related information (i.e., sex, age, purpose of travel, country of birth, and country of birth of the parents, travel duration, time between pre-travel consultation and departure date, visiting country) were obtained during the pre-travel consultation and registered in the electronic patient files. Country of birth of the participants and of their parents were categorized into low-, middle- and high-income country, following definitions from the World Bank Country and Lending Groups [16]. These variables were assessed as previous studies among travellers found an association between people with a migration background visiting friends and relatives and non-adherence to anti-malaria measures [4,[17], [18], [19], [20]]. Until September 13, 2021, all travellers were advised to bring SBET during travel. After the protocol changed, only travellers to remote areas where access to care was not possible within 48 hours were advised to bring SBET [9]. The reason for not prescribing SBET was noted in the electronic patient files. Atovaquone 250 mg and proguanil 100 mg was the most prescribed SBET (once daily 4 tablets during 3 days) [9].

### 2.2 Study design and data collection

Travellers aged ≥18 years, Dutch- or English-speaking, with an appointment at the travel clinic and the intention to travel ≤12 weeks to a moderate malaria risk area were asked during the consultation to participate in this prospective longitudinal study. Exclusion criteria were travelling to high-risk malaria areas and travelling without a mobile phone. Socio-demographic and travel-related data registered in the electronic patient files, were collected during pre-travel consultation, which also served as the pre-travel study visit. All participants were asked to download an application developed for the study on their mobile phone and to keep a daily diary during their trip until 14 days after their return. Through the diaries, information was collected on current location (selected country and area), use of mosquito repellents such as DEET, sleeping under an (impregnated) bed net and/or within an air-conditioned room, symptoms of disease, the use of SBET when having fever, seeking medical help, search for information on symptoms, diagnosis and medication used (Supplementary Table S1). Participants who did not complete the questionnaire for more than one day received a push notification as a reminder. After September 2021, the application was extended, and participants with fever were additionally asked whether they had carried SBET during travel. All participants were provided with a thermometer at the pre-travel study visit (Digitemp Electronic Thermometer, Servoprax GmbH, Wesel, Germany) and were asked to measure their temperature when feeling feverish. All participants were asked to donate a pre-travel blood sample, taken by a health care provider via venepuncture at the pre-travel study visit, and after return if they had experienced fever during travel. Blood samples (EDTA-blood) were centrifuged (Hettich Rotixa 50S, 10 min 3000 rpm) and frozen at −20 °C within 24 hours. Participants were provided with a dried blood spot card (Protein Saver 903 card, GE Healthcare, Cardiff, UK) and were instructed on how to self-collect blood during travel when experiencing fever, both in the application and on paper (Supplementary Table S1).

### 2.3 Statistical analysis

Travellers who did not complete the diary for more than six days during travel were excluded from the analysis to minimize the risk of missing any episode of fever. Age, sex and SBET prescription were compared between included and excluded participants, and non-included travellers visiting the Travel Clinic and travelling to moderate-risk areas using Pearson two sided chi-square test, Fisher two-sided exact test for categorical variables and the Kruskal-Wallis test for continuous variables. Continuous variables were summarised using the median, interquartile range (IQR), and range. Categorical variables were presented as numbers and percentages. The overlap between adherence to the various mosquito protection measures was visualized with a Venn diagram.

Adherence to mosquito protection measures was assessed through self-reported adherence, as recorded in the daily diaries. The following protection measures were assessed: (i) mosquito repellent use (DEET) during the evening/night, (ii) bed net use while sleeping, and (iii) air-conditioning use while sleeping. Adherence with mosquito protection measures was quantified by calculating the proportion of days adherent during the total days at risk of acquiring malaria, determined by the travel location, season, and in accordance with the LCR malaria maps during the travel period [9]. If the risk was unknown because information on exact location was missing, we assumed there was no risk of malaria. Participants were considered adherent if they reported to have used the mosquito protection measure ≥75 % of the days at risk of malaria.

We used Poisson regression with robust standard errors to assess determinants of adherence to mosquito protection measures. Prevalence ratio (PR) and the 95 % confidence interval (95 % CI) were calculated. Univariable regression analysis was used to assess the association between each independent variable (i.e., sex, age, purpose of travel, country of birth, and country of birth of the parents, travel duration, time between pre-travel consultation and departure, travel destination, and national LCR guideline applicable) and the outcome. The national LCR guideline applicable at the time of pre-travel consultation (i.e., SBET prescribed to all travellers per the 2017 guideline, or only to those visiting remote areas per the 2021 guideline) was included in the analysis as changes in recommendations regarding SBET prescription, may have influenced travellers’ awareness of malaria risk and, consequently, their adherence to anti-malaria measures. Variables with a p < 0.2 from a Wald χ^2^ test in univariable analysis were included in an initial multivariable model. Collinearity among the variables was assessed using the variance inflation factor (VIF). The final multivariable model was developed through a backwards selection process using the Akaike Information Criterion (AIC) and Bayesian Information Criterion (BIC).

Statistical significance was determined at a threshold of p < 0.05. Statistical analysis were performed using Stata (version 17.0; StataCorp College Station, TX).

### 2.4 Ethical approval

The study protocol was approved by the Medical Ethics Committee of the Amsterdam University Medical Centers location University of Amsterdam (MEC 2017_041). All participants provided written informed consent for participation.

## 3 Results

**Fig. 1:**
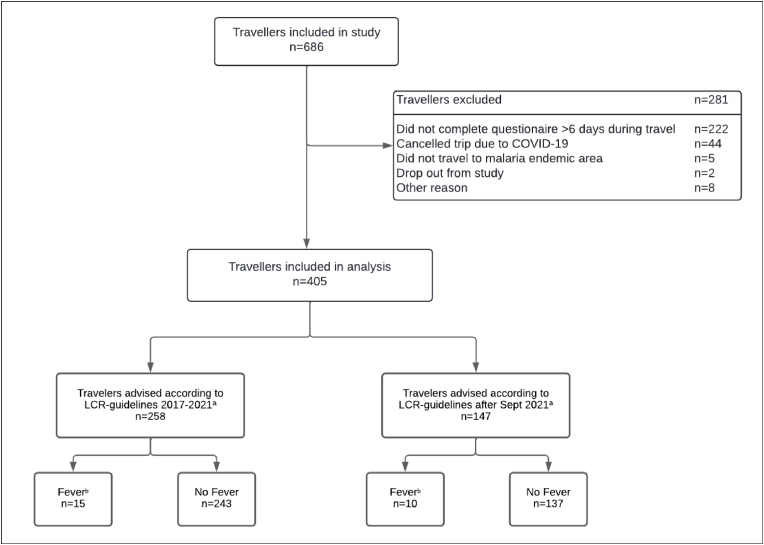
Flow chart of included participants in a prospective longitudinal study among short-term travellers to moderate malaria risk areas recruited at the Public Health Service of Amsterdam, Amsterdam, the Netherlands, 2018–2023. a. The Dutch Coordination Centre for Travellers' Health Advice (LCR) produces guidelines for travel doctors and nurses in the Netherlands. The guideline from 2017 until September 2021 stated that travellers should have an SBET when travelling to moderate malaria risk areas. The guidelines were updated in September 2021 and specify that only travellers to remote areas (where medical assistance cannot be reached <48 hours of fever onset) should have an SBET when travelling to moderate malaria risk areas. b. Fever measured using a provided thermometer, or feeling feverish in case temperature was not measured during travel up to 14 days after return.

**Fig. 2:**
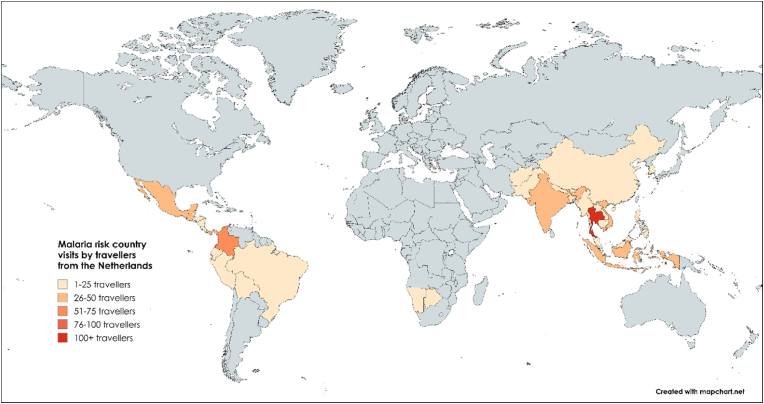
World map with visited malaria endemic countries by 405 travellers from the Netherlands participating in a prospective study at the Public Health Service of Amsterdam, Amsterdam, The Netherlands, between 2018 and 2023. Malaria endemic countries as defined by the Dutch Coordination Centre for Travellers' Health Advice (LCR) malaria maps at moment of pre-travel consultation. Map lines delineate study areas and do not necessarily depict accepted national boundaries.

Between October 15, 2018; and September 21, 2023, 686 travellers to moderate-risk malaria areas were recruited (Fig. 1). A total of 281 (41 %) participants were excluded from analyses: 222 participants did not complete the diaries >6 days during their travel; 44 participants cancelled their travel due to the COVID-19 pandemic; five participants did not travel to a malaria-endemic area, two participants dropped out before study conclusion, and eight participants were excluded because of other reasons. In total, 405 (59 %) participants were included in the analysis. Median age was 30 years (IQR 25–34), 241 (60 %) were female, 360 (89 %) were born in the Netherlands and 12 (3 %) were born in a low- or middle-income country (Table 1). The most frequently visited countries were Thailand (n = 105; 17 %), Indonesia (n = 76; 12 %), and Colombia (n = 67; 11 %) (Table 1, Fig. 2) and the median travel duration was 23 days (IQR 17–33). The main purpose of travel was tourism (n = 384; 95 %). Median time from pre-travel consultation until departure was 23 days (IQR 11–39). In total, 258 (64 %) participants were advised according to the national LCR guideline of 2017 and 147 (36 %) according to the guideline of 2021 (Fig. 1). The included participants were comparable to the excluded participants and non-included travellers to moderate risk malaria areas visiting the pre-travel clinic regarding age, sex and amount of SBET prescriptions (Supplementary Table S2).

**Table 1:** Characteristics of 405 travellers to moderate-risk malaria areas who participated in a prospective longitudinal study among travellers at the Public Health Service of Amsterdam, Amsterdam, the Netherlands, between 2018 and 2023.

Characteristic of participants	n (%)^a^
**Sex**	
Male	164 (40.5 %)
Female	241 (59.5 %)

**Age** (years)	
Median [IQR] (range)	30 [25–34] (18–81)
18 to <30 years	198 (48.9 %)
30 to <40 years	145 (35.8 %)
≥40 years	62 (15.3 %)

**Region of birth**^b^	
The Netherlands	360 (88.9 %)
High-income country (excluding the Netherlands)	31 (7.7 %)
Low/Middle-income country	12 (3.0 %)
Unknown	2 (0.5 %)

**Region of birth of parents**^b^^,^^c^	
The Netherlands	300 (74.1 %)
High-income country (excluding the Netherlands)	58 (14.3 %)
Low/Middle-income country	44 (10.9 %)
Unknown	3 (0.7 %)

**Countries visited during travel period**^d^	
Thailand	105 (17.1 %)
Indonesia	76 (12.4 %)
Colombia	67 (10.9 %)
Guatemala	39 (6.3 %)
India	35 (5.7 %)
Mexico	28 (4.6 %)
Panama	26 (4.2 %)
Vietnam	26 (4.2 %)
Ecuador	24 (3.9 %)
Philippines	24 (3.9 %)
Other	164 (26.8 %)

**Travel duration**^e^	
Median (days) [IQR] (range)	23 [17–33] (4–91)
<3 weeks	143 (35.3 %)
3 weeks to <1 month	146 (36.1 %)
1 to <2 months	87 (21.5 %)
2 to <3 months	29 (7.2 %)

**Purpose of travel**	
Tourism	384 (94.8 %)
Work/education	8 (2.0 %)
Visiting friends and relatives	13 (3.2 %)

**Time from pre-travel consultation until departure**	
Median (days) [IQR] (range)	23 [11–39] (0–141)
<1 week	59 (14.6 %)
1 to <3 weeks	127 (31.4 %)
3 to <5 weeks	91 (22.5 %)
5 to <7 weeks	68 (16.8 %)
≥7 weeks	60 (14.8 %)

IQR = interquartile range.

a Unless otherwise indicated.

b Countries categorized by income levels for 2024 by the World Bank Country and Lending Groups [16].

c Region of birth of parents was determined by the birth country of the parent from the lowest income country.

d Visited malaria endemic countries according to the Dutch Coordination Centre for Travellers' Health Advice (LCR) [9]. A participant could have visited multiple countries; thus, the total percentage does not add up to 100 %.

e The travel duration from departure to return was limited to a maximum of 3 months based on inclusion criteria (i.e., short-term travel).

Of the 405 participants, 290 (72 %) received an SBET prescription during the pre-travel consultation (Table 2). According to the LCR guideline from 2017, travellers to moderate-risk areas were advised to carry SBET. Overall, 88 % (n = 226/258) of participants received both a recommendation to use SBET and an SBET prescription. The remaining 12 % (n = 32) were also advised to use SBET, but for various reasons did not receive a prescription. These reasons were registered in the electronic patient files and included: participants preferring to arrange SBET independently (n = 13; 41 %), participants already in possession of SBET (e.g. from previous travel, n = 12; 38 %), participant sharing a prescription with a fellow traveller (n = 1; 3 %) and participants where the reason for not prescribing was unknown (n = 6; 19 %). Following the implementation of the LCR guideline of September 2021, only travellers to remote areas were recommended to carry SBET. During this period, 44 % (n = 64/147) of participants received both a recommendation and an SBET prescription. Among the 56 % (n = 83) who were not prescribed SBET, the reasons were as follows: participants did not intend to travel remotely (n = 74; 89 %), participants already in possession of SBET (n = 2; 2 %), participants sharing a prescription with a fellow traveller (n = 6; 7 %) and participants where the reason for not prescribing was unknown (n = 1; 1 %).

**Table 2:** Adherence to fever-related advices (SBET use and medical doctor visit) according to the Dutch LCR guidelines and symptoms during travel of 405 participants of a prospective longitudinal study, Amsterdam, the Netherlands, between 2018 and 2023.

Characteristics	Total (n = 405)	Guideline 2017 until 2021^a^ (n = 258)	Guideline from 2021^a^ (n = 147)
	n (%)	n (%)	n (%)
**SBET prescribed during pre-travel consultation**			
Yes	290 (71.6 %)	226 (87.6 %)	64 (43.5 %)
No	115 (28.4 %)	32 (12.4 %)	83 (56.5 %)

**Symptoms reported during travel**			
Yes	134 (33.1 %)	86 (33.3 %)	48 (32.7 %)
No	271 (66.9 %)	172 (66.7 %)	99 (67.3 %)

Characteristics in case of symptoms	Total participants with symptoms (n = 134)	Total participants with symptoms (n = 86)	Total participants with symptoms (n = 48)
**Symptoms reported**^b^			
Headache	76 (56.7 %)	45 (52.3 %)	31 (64.6 %)
Nausea	62 (46.3 %)	42 (48.8 %)	20 (41.7 %)
Vomiting	30 (22.4 %)	18 (20.9 %)	12 (25.0 %)
Diarrhoea (at least 3 times diarrhoea/day)	71 (53.0 %)	44 (51.2 %)	27 (56.3 %)
Muscle ache (other than after activity)	37 (27.6 %)	28 (32.6 %)	9 (18.8 %)
Fever^e^	25 (6.2 %)	15 (5.8 %)	10 (7.3 %)
Other (e.g. coughing, abdominal pain, fatigue)	67 (50.0 %)	43 (50.0 %)	24 (50.0 %)

**Searched for information in case of symptoms**			
Yes^c^	58 (43.3 %)	32 (37.2 %)	26 (54.2 %)
Online	58 (100 %)	32 (100 %)	26 (100 %)
Email contact with the travel clinic	2 (3.4 %)	1 (3.1 %)	1 (3.8 %)
Brochure received during pre-travel consultation	1 (1.7 %)	1 (3.1 %)	0 (0.0 %)
Insurance company	1 (1.7 %)	0 (0.0 %)	1 (3.8 %)
Local pharmacy	1 (1.7 %)	0 (0.0 %)	1 (3.8 %)
Other (fellow travellers, locals)	0 (0.0 %)	0 (0.0 %)	0 (0.0 %)
No	76 (56.7 %)	54 (62.8 %)	22 (45.8 %)

**Treatment taken (other than SBET)**			
Yes^d^	26 (19.4 %)	17 (19.8 %)	9 (18.8 %)
Painkillers (paracetamol or NSAIDs)	12 (46.2 %)	5 (29.4 %)	7 (77.8 %)
Antidiarrheal (Loperamide, activated charcoal)	17 (65.4 %)	10 (58.8 %)	7 (77.8 %)
Oral Rehydration Solution (ORS)	2 (7.7 %)	0 (0.0 %)	2 (22.2 %)
Antibiotics	4 (15.4 %)	2 (11.8 %)	2 (22.2 %)
Other (e.g. laxantia, anti-nausea, nose spray)	7 (26.9 %)	4 (23.5 %)	3 (33.3 %)
No	108 (80.6 %)	69 (80.2 %)	39 (81.3 %)

**Activities postponed due to symptoms**			
Yes	52 (38.8 %)	36 (41.9 %)	16 (33.3 %)
No	82 (61.2 %)	50 (58.1 %)	32 (66.7 %)

**Changes in travel plans due to symptoms**			
Yes	21 (15.7 %)	14 (16.3 %)	7 (14.6 %)
No	113 (84.3 %)	72 (83.7 %)	41 (85.4 %)

Characteristics in case of fever	Participants with fever (n = 25)	Participants with fever (n = 15)	Participants with fever (n = 10)
**Start of fever**^e^			
<7 days after arrival^f^	5 (20.0 %)	4 (26.7 %)	1 (10.0 %)
≥7 days after arrival until date of return	14 (56.0 %)	8 (53.3 %)	6 (60.0 %)
Within 2 weeks after return	6 (24.0 %)	3 (20.0 %)	3 (30.0 %)

**Doctor visit**^e^			
Yes	5 (20.0 %)	3 (25.0 %)	2 (20.0 %)
<7 days after arrival^f^	1 (20.0 %)	1 (33.3 %)	0 (0.0 %)
≥7 days after arrival until date of return	3 (60.0 %)	2 (66.6 %)	1 (50.0 %)
Within 2 weeks after return	1 (20.0 %)	0 (0.0 %)	1 (50.0 %)
No	20 (80.0 %)	12 (75.0 %)	8 (80.0 %)

**Used SBET**			
Yes	0 (0.0 %)	0 (0.0 %)	0 (0.0 %)
No	25 (100.0 %)	15 (100.0 %)	10 (100.0 %)

**Study procedure: Provided self-sampled dried blood spot collected during travel**			
Yes	4 (16.0 %)	2 (13.3 %)	2 (20.0 %)
No	21 (84.0 %)	13 (86.7 %)	8 (80.0 %)
**Study procedure: Blood sample was obtained after return**^g^			
Yes	19 (76.0 %)	11 (73.3 %)	8 (80.0 %)
No	6 (24.0 %)	4 (26.7 %)	2 (20.0 %)

**Abbreviations**: LCR = Dutch Coordination Centre for Travellers' Health Advice, NSAIDs = non-steroidal anti-inflammatory drugs, ORS = oral rehydration solution, SBET= standby emergency treatment.

a The Dutch Coordination Centre for Travellers' Health Advice (LCR) produces guidelines for travel doctors and nurses in the Netherlands. The guideline from 2017 until September 2021 stated that travellers should have an SBET when travelling to moderate malaria-endemic areas. The guidelines were updated in September 2021 and specify that only travellers to remote areas (where medical assistance cannot be reached <48 hours of fever onset) should have an SBET when travelling to moderate malaria-endemic areas.

b Percentage of participants with symptoms may not sum up to 100 % as participants may report multiple symptoms.

c Percentage of participants searching for information may not sum up to 100 % as multiple information sources could be used by participants.

d Percentage of participants using a treatment other than SBET in case of symptoms may not add up to 100 % as participants may have used multiple treatment options.

e Fever measured using a provided thermometer, or feeling feverish in case temperature was not measured during travel up to 14 days after return.

f As the minimum incubation period for malaria is 7 days, presentation of fever within the first 7 days of travel may not be attributed to malaria acquired during this travel.

g Participants with fever during travel or within 14 days after return were asked to donate a second blood sample at the Public Health Service of Amsterdam after returning to the Netherlands.

In total, 134 (33 %) participants reported experiencing symptoms either during travel or within 14 days of their return (Table 2). The most-prevalent symptoms were headache (n = 76; 57 %) and gastrointestinal symptoms (n = 94; 70 %), including nausea (n = 62; 46 %), vomiting (n = 30; 22 %), and diarrhoea (n = 71; 53 %). Due to these symptoms, 52 (39 %) postponed activities and 21 (16 %) altered their travel plans. Among those who experienced symptoms, 58 (43 %) sought information online about their symptoms, two (1 %) emailed the pre-travel clinic, one (1 %) used an information brochure of the LCR received during the pre-travel consultation, and one (1 %) visited a local pharmacy.

### 3.1 SBET utilization

Of the 405 participants, 25 (6 %) experienced fever, of whom 5/25 (20 %) developed fever within seven days of arrival at their travel destination and 6/25 (24 %) within 14 days after return (Table 2, Supplementary Table S3). Out of the 25 febrile travellers, 18 (72 %) had received SBET prescription during pre-travel consultation, albeit none used SBET when they experienced fever and only 5 (20 %) attended a local health care facility. The duration of fever ranged from one to three days (Supplementary Table S3). None of the participants used anti-malarial medication or were diagnosed with malaria. Furthermore, none of the travellers without fever used SBET. Among the 25 travellers who reported fever, 19 (76 %) provided a blood sample after their return, and 4 (16 %) provided a self-collected dried blood spot.

### 3.2 Adherence to mosquito protection measures

Detailed information on malaria risk for each travel day was available for 356 (out of 405, 88 %) participants, covering a total of 15,195 days, with 6,351 (42 %) of those days classified as at-risk for malaria. Of these 356 participants, 342 (96 %) reported using mosquito repellent at least once during their travel, with DEET being the most-commonly reported (n = 328; 92 % participants) (Table 3). Of 328 DEET users, 309 (94 %) reported using it during the night/evening. In total 123 (40 %; 35 % of the total 356 participants) reported applying it during the evening or night ≥75 % of the days they were at-risk and were thus considered to be adherent. In multivariable analysis, participants who travelled 1–3 months compared to travelling <3 weeks (PR 0.58; 95 % CI 0.39–0.87) were less likely to be adherent to use DEET during the evening and night (Table 4).

**Table 3:** Adherence to mosquito protection measures among 356 travellers at risk of malaria travelling to moderate risk malaria areas.

Mosquito protection measure during travel	All travellers^a^ (n = 356)	Traveller with fever^b^ (n = 22)	Travellers without fever^b^ (n = 334)
	**n (%)**	**n (%)**	**n (%)**
**Repellant used (during ≥1 day or night)**			
Yes	342 (96 %)	21 (95 %)	321 (96 %)
Only DEET use	259 (76 %)	16 (76 %)	243 (76 %)
Only other repellent (e.g. Icaridin) use	14 (4 %)	0 (0 %)	14 (4 %)
DEET and other repellent used	69 (20 %)	5 (24 %)	64 (20 %)
No	14 (4 %)	1 (5 %)	13 (4 %)

**Highest DEET percentage used**^c^			
<40 % DEET	36 (10 %)	3 (14 %)	33 (10 %)
40–50 % DEET	209 (59 %)	13 (59 %)	196 (59 %)
>50 % DEET	80 (22 %)	5 (23 %)	75 (22 %)
% DEET missing	3 (1 %)	0 (0 %)	3 (1 %)
No DEET used	28 (8 %)	1 (5 %)	27 (8 %)

**DEET use in the evening/night**^c^, ^d^			
Yes	309 (87 %)	19 (86 %)	290 (87 %)
<25 % of days at risk	46 (15 %)	4 (21 %)	42 (14 %)
25–75 % of days at risk	140 (45 %)	8 (42 %)	132 (46 %)
≥75 % of days at risk	123 (40 %)	7 (37 %)	116 (40 %)
No	47 (13 %)	3 (14 %)	44 (13 %)

**Mosquito bed net use at night**^d^			
Yes	206 (58 %)	16 (73 %)	190 (57 %)
<25 % of days at risk	90 (44 %)	10 (63 %)	80 (42 %)
25–75 % of days at risk	97 (47 %)	4 (25 %)	93 (49 %)
≥75 % of days at risk	19 (9 %)	2 (13 %)	17 (9 %)
No	150 (42 %)	6 (27 %)	144 (43 %)

**Air-conditioning use at night**^d^			
Yes	320 (90 %)	19 (86 %)	301 (90 %)
<25 % of days at risk	54 (17 %)	5 (26 %)	49 (16 %)
25–75 % of days at risk	137 (43 %)	6 (32 %)	131 (44 %)
≥75 % of days at risk	129 (40 %)	8 (42 %)	121 (40 %)
No	36 (10 %)	3 (14 %)	33 (10 %)

Abbreviations: DEET = N,N-diethyl-3-methylbenzamide, LCR = Dutch Coordination Centre for Travellers' Health Advice.

a Travellers at risk for contracting malaria (low- or moderate-risk) recorded daily in a mobile application during travel, based on the participant's location and the presence of malaria in that region, as indicated by the malaria maps of the LCR. Only participants with days where the risk of malaria was known were included.

b Fever measured using a provided thermometer, or feeling feverish in case temperature was not measured during travel up to 14 days after return.

c According to the Dutch LCR guidelines a maximum of 50 % DEET is advised during the evening/night to protect against mosquitos in non-pregnant travellers above the age of 2 years old [9].

d Number of days with mosquito protection measure against malaria divided by the total number of travel days with malaria risk.

**Table 4:** Determinants of adherence to DEET use^a^ aimed at reducing malaria risk in travellers at risk of malaria (n = 356); univariable and multivariable Poisson regression analysis.

Characteristic	Participants with malaria risk during travel		Univariable regression			Multivariable regression^h^		
	Non-adherent to DEET use^a^ N = 233	Adherent to DEET use^a^ N = 123	PR	95 % CI	p-value	PR	95 % CI	p-value
**Sex**								
Female	133 (57 %)	81 (66 %)	REF					
Male	100 (43 %)	42 (34 %)	0.78	(0.57–1.06)	0.115			

**Age**								
<29 year	112 (48 %)	62 (50 %)	REF					
30–39 year	78 (33 %)	49 (40 %)	1.08	(0.80–1.46)				
≥40 year	43 (18 %)	12 (10 %)	0.61	(0.36–1.05)	0.124			

**Country of birth**								
Netherlands	205 (88 %)	112 (91 %)						
Other countries	26 (11 %)	11 (9 %)	REF					
Unknown	2 (1 %)	0 (0 %)	0.84	(0.50–1.41)	0.514			

**Country of birth of parents**^b^^,^^c^								
Low/Middle-income country	25 (11 %)	13 (11 %)						
High-income country (excl. Netherlands)	31 (13 %)	15 (12 %)	REF					
Netherlands	175 (75 %)	94 (76 %)	0.95	(0.52–1.75)				
Unknown	2 (1 %)	1 (1 %)	1.02	(0.64–1.64)	0.954			

**Time from pre-travel consultation until departure**								
<2 week	71 (30 %)	36 (29 %)	REF					
2–5 weeks	84 (36 %)	48 (39 %)	1.08	(0.76–1.53)				
≥5 weeks	78 (33 %)	39 (32 %)	0.99	(0.68–1.43)	0.857			

**National LCR guideline**^d^								
Guideline from 2017	142 (61 %)	81 (66 %)	REF					
Revised guideline implemented in 2021	91 (39 %)	42 (34 %)	0.87	(0.64–1.18)	0.369			

**Reason for travel**								
Tourism	218 (94 %)	119 (97 %)	REF					
Other travel reason (work, education, visiting friends and relatives)	15 (6 %)	4 (3 %)	0.60	(0.25–1.44)	0.252			

**Travel duration**								
<3 weeks	68 (29 %)	48 (39 %)	REF			REF		
3 weeks–1 month	83 (36 %)	49 (40 %)	0.90	(0.66–1.22)		0.90	(0.65–1.22)	0.493
1–3 months	82 (35 %)	26 (21 %)	0.58	(0.39–0.87)	0.028	0.58	(0.39–0.87)	0.008

**Travel destination**^e^^f^								
Asia	148 (64 %)	82 (67 %)	REF					
Latin America	85 (36 %)	40 (33 %)	0.90					
Africa	0 (0 %)	1 (1 %)	f	(0.66–1.22)	0.494			

**SBET prescribed**								
No	70 (30 %)	30 (24 %)	REF					
Yes	163 (70 %)	93 (76 %)	1.21	(0.86–1.70)	0.271			

**Symptoms during travel**								
No	150 (64 %)	84 (68 %)	REF					
Yes	83 (36 %)	39 (32 %)	0.89	(0.65–1.22)	0.465			

**Fever during travel**^g^								
No	218 (94 %)	116 (94 %)	REF					
Yes	15 (6 %)	7 (6 %)	0.92	(0.49–1.72)	0.785			

**Abbreviations**: 95 % CI = 95 % Confidence Interval, DEET = N,N-diethyl-3-methylbenzamide, LCR = Dutch Coordination Centre for Travellers' Health Advice, PR=Prevalence Ratio, REF = reference group, SBET= standby emergency treatment.

Due to limited numbers in the ‘unknown’ category, unknown was not used in the analyses. Sum can be more or less than 100 % due to rounding.

a DEET use was assessed by calculating the percentage of days DEET was applied during the evening/night divided by the total travel days with malaria risk. Adherence was categorized as follows: <75 % use of DEET was classified as non-adherent, ≥75 % use was classified as adherent.

b Countries categorized by income levels for 2024 by the World Bank Country and Lending Groups [16].

c Region of birth of parents was determined by the birth country of the parent from the lowest income country.

d The Dutch Coordination Centre for Travellers' Health Advice (LCR) produces guidelines for travel doctors and nurses in the Netherlands. The guideline from 2017 until September 2021 stated that travellers should have an SBET when travelling to moderate malaria-endemic areas. The guidelines were updated in September 2021 and specify that only travellers to remote areas (where medical assistance cannot be reached <48 hours of fever onset) should have an SBET when travelling to moderate malaria-endemic areas [9].

e If a participant visited multiple continents with malaria risk, the continent where the participant spend the most days was included.

f As only one participant visited Africa, this category was excluded from the Poisson regression analysis.

g Fever measured using a provided thermometer, or feeling feverish during travel up to 14 days after return.

h Variable included in the initial multivariable Poisson regression model: travel duration.

Mosquito bed nets were infrequently used by participants to moderate malaria risk areas, with 206 (58 %) participants reporting usage at least once during their travel and 19 (9 %; 5 % of the total 356 participants) reporting to have used a bed net ≥75 % of time at-risk (Table 3). Multivariable regression analysis showed no significant determinants of bed net use (Supplementary Table S4). Sleeping in an air-conditioned room was reported by 320 (90 %) of participants at least once during their travel, with 129 (40 %, 36 % of the total 356 participants) using it ≥75 % of days at-risk (Table 3). In multivariable analysis, participants with destination in Latin America were less likely to be adherent to air-conditioning use compared to Asia (PR 0.34; 95 % CI 0.22–0.51) (Supplementary Table S5).

**Fig. 3:**
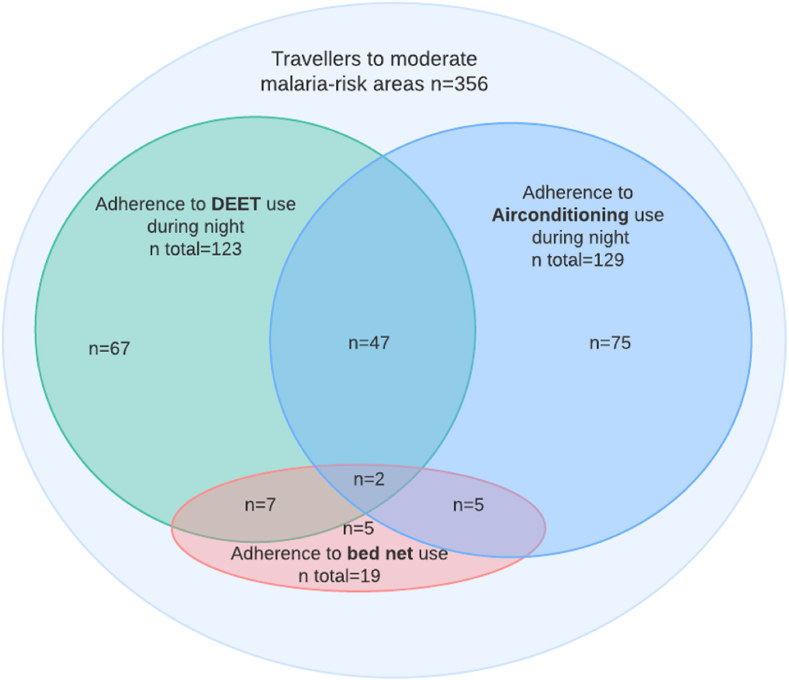
**Association between adherence to various mosquito protection measures among travellers at risk of malaria travelling to moderate risk regions**. Venn diagram of various mosquito protection measures demonstrating their relation. A participant was considered adherent to the mosquito protection measure when they reported to have used the measure ≥75 % of the time when at risk of malaria.

Among the participants at-risk, 208 (58 %) were adherent to at least one anti-mosquito measure to prevent malaria ≥75 % of the time at-risk. Sixty-one participants (29 % of 208 adherent) used at least two measures, with DEET use and sleeping in an air-conditioned room being the most frequently combined (Fig. 3).

## 4 Discussion

In this prospective longitudinal study among travellers to moderate-risk malaria areas, we found that 6 % of participants reported fever during their travel or within 14 days after return. None of those with fever used the prescribed SBET and only 20 % attended a local health care facility. Adherence to mosquito protection measures was poor, despite pre-travel counselling. Only 35 % of participants used DEET and 5 % used a bed net on ≥75 % of the nights they were at-risk of malaria.

Only four of the 25 participants complied with the study procedure of self-collecting a dried blood spot sample when experiencing a fever. The exact reason why the other travellers with fever did not collect a sample is unknown; but may be related to feeling too unwell to perform the self-collection; not considering malaria as a possible cause of their symptoms; or forgetting to bring the required materials. We included this measure as we aimed to test these spots for Plasmodium infection after return, but the number of dried blood spots was too small to do so. In addition, we initially aimed to test for *Plasmodium* spp. infection by detecting anti-*P. falciparum* circumsporozoite protein (*Pf*CSP) antibodies in the blood samples obtained by venepuncture after return to estimate the incidence of *P. falciparum* exposure. However, several studies conducted during our study period reported very limited sensitivity of the latter method, likely due to the brief time period between sporozoite inoculation and hepatic invasion (the life-cycle stage at which *Pf*CSP is exposed), as well as the very small number of sporozoites (hence a very limited amount of antigen presented) transmitted during naturally acquired malaria infection [21]. Consequently, we concluded that testing the blood samples would not be of additional value for this research although the proportion of febrile participants with a blood sample was 76 % (19/25). It is unlikely that the 25 febrile participants contracted malaria. The fever in these participants resolved within a maximum of three days without malaria treatment, rendering a *P. falciparum* infection very unlikely, as malaria is generally not self-limiting in a non-immune person [21].

Since the implementation of the revised national LCR guideline in September 2021, which recommends prescribing SBET only for travellers visiting remote areas, the proportion of travellers receiving a prescription has decreased from 88 % to 44 %. Based on a study on travel times to healthcare facilities with motorized transportation, most regions visited by travellers in our study were accessible within a day [22]. Therefore, a 44 % prescription rate for SBET is high and may be due to undecided travel plans during the pre-travel consultation or change in travel destinations. To reduce costs and medication spillage, we believe travellers who require SBET should be more carefully selected during pre-travel consultation.

Like our results, two systematic reviews showed that SBET use was very low among travellers, although in these reviews still 2 % of travellers reported using SBET [13,14]. Mild symptoms and the short duration of fever reported by our participants may be a reason for not using SBET. This might also explain that the majority did not seek medical care. However, qualitative research is needed to gain a deeper understanding of the considerations behind these decisions. A systematic review demonstrated that most travellers are capable of self-administering self- or doctor-initiated rapid diagnostic tests (RDTs) to confirm malaria infection before using SBET [23]. Given the increased familiarity with self-testing due to the COVID-19 pandemic, exploring this strategy further for a specific group of travellers to remote areas could potentially enhance adherence.

Our study found low adherence to mosquito protection measures, like findings in other studies [20,[24], [25], [26]]. Plausible explanations for low adherence may include travellers’ perception of a low risk of malaria exposure, the absence or lack of bed nets, failure to notice mosquitoes, inadequate pre-travel counselling due to information overload or insufficient attention to mosquito protection measures. The low number of participants born in low- and middle-income countries may have contributed to a higher adherence to mosquito protection measures, as literature indicates that visiting friends and relatives is associated with lower adherence [4,[17], [18], [19], [20]]. Longer travel duration was associated with lower adherence to DEET use, which is consistent with other studies and may have to do with fatigue of regularly applying DEET over a longer period of time [20,24,25]. However, these measures are important for preventing malaria, particularly in low- and moderate-risk areas where chemoprophylaxis is not used [24,25]. Therefore, these measures should receive sufficient attention during pre-travel consultation.

Although not all travellers may have been aware of the anti-mosquito effect of air-conditioning, a substantial 36 % reported sleeping in an air-conditioned room for ≥75 % of the days they were at risk. Travel to Asia was associated with higher adherence to air-conditioning recommendations compared to travel to South America, similar to a previous study [20]. This increased adherence in Asia may be due to the region's higher temperatures, more humid climate and greater availability of air-conditioned accommodations. A proportion of travellers who were less compliant to DEET use were also less compliant to air-conditioning and bed net use recommendations. As suggested in previous research, personality traits may play a significant role in malaria prevention, rather than travellers consciously selecting between different preventive measures to reduce the risk [20,21].

The strength of this study lies in the prospective longitudinal data collection with detailed information per travel day, bringing the data close to real-life conditions. Our study also has some limitations. First, most data were collected through self-reported daily diaries to reduce recall bias, but this may have led to socially desirable behaviour, resulting in a possible overestimation of adherence estimates. Second, the COVID-19 pandemic during the study period may have heightened participants’ awareness of infectious disease symptoms and influenced their behaviour, resulting in a possible overestimation of adherence estimates. Moreover, conducting the study during a period of the pandemic may have resulted in the inclusion of participants who are more adventurous and less likely to follow the advice during travels, or just after the travel restrictions than in non-pandemic study periods, resulting in a possible underestimation of adherence estimates. Furthermore, wearing protective clothing was not explored in the questionnaire, which may be of interest for future research.

Travellers included in this study were young, with a median age of 30 years and the majority went travelling for touristic reasons (95 %). These characteristics are comparable to those observed in other studies [3,15,21] and other travellers visiting the Travel Clinic at the Public Health Service in Amsterdam, supporting the assumption that the current findings can be generalised to other traveller populations attending pre-travel clinics and travelling to moderate malaria risk areas. The absence of malaria cases in our study is most likely explained by the low incidence of malaria in moderate-risk areas, defined as regions with an annual falciparum index of ≤10 cases per 1000 local population [7,9].

In conclusion, our findings show that SBET is not used by travellers to moderate malaria-risk areas in the event of fever, and only a minority seek medical help to rule out malaria. Furthermore, although almost half of travellers received SBET prescription during pre-travel advice, they presumably did not travel to remote areas. Therefore, travellers with need of SBET should be more carefully selected to reduce costs and medication spillage. Further research should focus on better understanding the behavioural concepts underlying the use of SBET and malaria preventive measure and investigate the use of traveller initiated RDTs for malaria diagnosis to improve travellers adherence.

## CRediT authorship contribution statement

**Daniel Julien Franken:** Writing – original draft, Resources, Project administration, Formal analysis, Conceptualization. **Vita Willemijn Jongen:** Writing – review & editing, Supervision, Methodology, Formal analysis. **Anna Rooyakkers:** Formal analysis. **Martin Peter Grobusch:** Writing – review & editing, Supervision. **Jelte Elsinga:** Writing – review & editing. **Margarita Boering:** Writing – review & editing. **Maria Prins:** Writing – review & editing, Supervision, Resources, Methodology, Formal analysis, Conceptualization. **Brigitte Antonia Geertruida Lucía van Cleef:** Writing – review & editing, Supervision, Methodology, Investigation, Formal analysis.

## Disclosure

None of the authors has declared any conflicts of interest.

## Funding sources

This work was supported by the Ministry of Health, Welfare and Sport of the Dutch Government within the program ‘Strengthening infectious disease control and pandemic preparedness of the regional Public Health Services’ in 2023–2024. This project was also supported by the Research & Development grants from the Public Health Service of Amsterdam, the Netherlands.

## Declaration of competing interest

The authors declare the following financial interests/personal relationships which may be considered as potential competing interests: Daniel Franken reports financial support was provided by Netherlands Ministry of Health Welfare and Sport. If there are other authors, they declare that they have no known competing financial interests or personal relationships that could have appeared to influence the work reported in this paper.
