# Supplementary material for: Adherence to stand-by emergency treatment and mosquito protection measures in short-term travellers to moderate malaria risk areas
Source: New Microbes New Infect. 2025 Jan 1;63:101561. doi: 10.1016/j.nmni.2024.101561 (PMC11840869; doi:10.1016/j.nmni.2024.101561)

Supplementary Table S1. Questionnaire during travel until 14 days after return.

Nr.	Question				Outcome
1	What is your departure/return date?				Date
2	In which country are you now?				Select country/area[a]
Preventive measures					
3	Did you use DEET today?				Yes/No
	3a	If so, did you use DEET during the day, in the evening or both			Daytime/Evening/Both
	3b	What percentage did you use?			<40%/40-50%/>50%
	3c	If No: Did you use a different repellent?			Yes/No
		3d	If yes: Which?		Open
4	Did you sleep under a bed net today?				Yes/No
5	Did you sleep in a closed, air-conditioned room today?				Yes/No
Symptoms					
6	Did you have any symptoms in the past 24 hours?				Yes/No
	6a	If Yes: Have you had a fever (over 38.5 degrees Celsius) and/or cold shivers?			Yes/No
		6b	What was the highest measured temperature?		Open
		6c	How did you measure it? (Oral, rectal or under the armpit)		Rectal/Oral/Under the armpit
	6d	Have you had a headache?			Yes/No
	6e	Have you had nausea?			Yes/No
	6f	Have you vomited?			Yes/No
	6g	Have you had diarrhoea? (At least 3x/day watery diarrhoea)			Yes/No
	6h	Have you had muscle ache? (Except after exercise)			Yes/No
	6i	Have you had any other symptoms than those mentioned above?			Open
Actions					
7	Have you searched for information about your symptoms?				Yes/No
	7a	How did you do this? (online, brochure, travel clinic, other)			Online/Brochure/Travel clinic?/Other?
		7b	If other: How did you do this?		Open
	7c	Did you seek medical advice?			Yes/No
		7d	If yes: From whom did you get advice? (fellow travellers, locals, insurance company, local pharmacy, other?)		Open
		7e	What advice did you get?		Open
	7f	Have you visited a hospital or doctor?			Yes/No
	7g	Have you taken any treatment?			Yes/No
		7h	If yes: did you take the stand-by treatment?		Yes/No
		7i	If yes: did you take other medication than you are used to?		Yes/No
			7j	If yes: which medication did you take?	Open
	7k	Did you get a diagnosis?			Yes/No
		7l	If yes: What was the diagnosis?		Open
	7m	Did you change your travel plans because of your symptoms?			Yes/No
	7n	Did you have to delay activities because of your symptoms?			Yes/No
Questions after return					
8	Did you bring your stand by treatment during your trip?				Yes/No[b]
9	Did you bring your bloodspot during your trip?				Yes/No[b]
10	Did you have any symptoms in the past 24 hours?<br>If yes go to symptoms				Yes/No

a. Selectable areas within a country were based on malaria risk areas indicated on the malaria maps, enabling identification of the participants risk at the time of completing the questionnaire.
b. Question asked once after return from travel in case of fever and after the implementation of the LCR guideline of September 2021.

Print screens of questionnaire in the app developed for data collection of this longitudinal prospective study

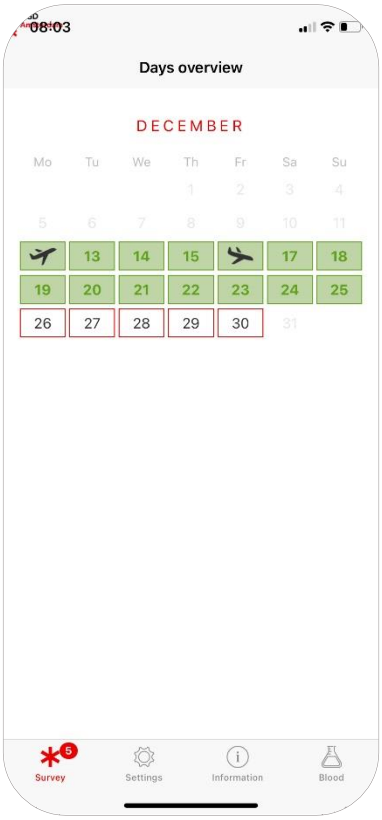

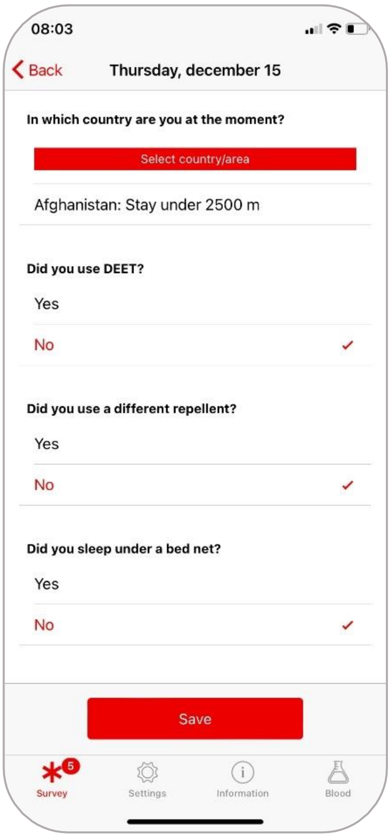

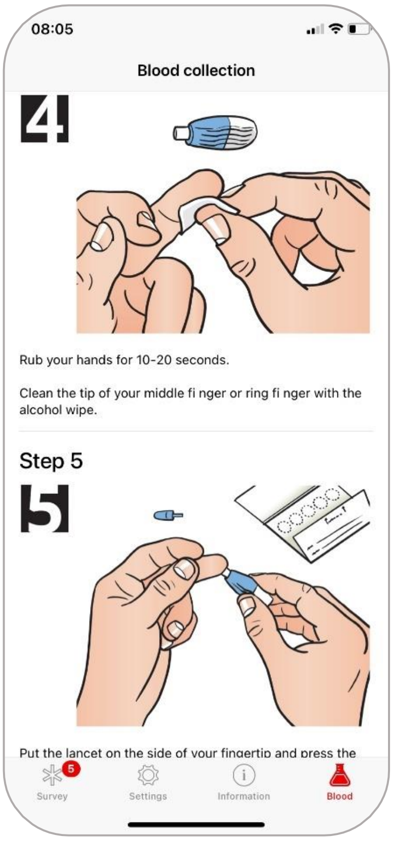

Supplement: Multimedia component 1 [file mmc1.pdf]
