# Supplementary material for: Adherence to stand-by emergency treatment and mosquito protection measures in short-term travellers to moderate malaria risk areas
Source: New Microbes New Infect. 2025 Jan 1;63:101561. doi: 10.1016/j.nmni.2024.101561 (PMC11840869; doi:10.1016/j.nmni.2024.101561)
Supplement: Multimedia component 2 [file mmc2.pdf]

Supplementary Table S2. Characteristics of 405 included participants, 277 excluded participants and 3,716 non-included travellers attending the pre-travel clinic of the Public Health Service of Amsterdam and travelling to moderate malaria risk areas.

**Characteristic of participants**	Included participants n=405	Excluded participants[b] n=277	Non-included travellers[c] n=3,716	P-value[d]
	n (%)[a]	n (%)[a]	n (%)[a]	
**Sex**				0.12
Male	164 (40%)	115 (42 %)	1,682 (45%)	
Female	241 (60%)	160 (58%)	2,034 (55%)	
**Age** (years)				<0.001
Median [IQR]	30 [25-34]	27 [23-32]	30 [26-35]	
**SBET prescribed during pre-travel consultation according to LCR guideline from 2017**	n=258	n=186	N.A.	0.75
No	32 (12%)	25 (13%)		
Yes	226 (88%)	161 (87%)		
Unknown	0 (0.0%)	0 (0.0%)		
**SBET prescribed during pre-travel consultation according to LCR guideline from 2021**	n=147	n=91	n=3,811	0.12
No	83 (57%)	54 (59%)	2,390 (64%)	
Yes	64 (44%)	35 (38%)	1,326 (36%)	
Unknown	0 (0.0%)	2 (2%)	0 (0%)	

IQR=interquartile range, N.A.=not applicable, SBET=standby emergency treatment.

a. Unless otherwise indicated.

b. Four excluded participants are missing in data because no research number was created for these participants.

c. Non-included travellers are travellers attending the Public Health Service of Amsterdam for pre-travel advice and travelling to moderate malaria-risk areas in the year 2022 and 2023.

d. P-values are based on the Pearson two-sided chi-square test or Fisher two-sided exact test for categorical variables and the Kruskal-Wallis test for continuous variables.
