# Supplementary material for: Adherence to stand-by emergency treatment and mosquito protection measures in short-term travellers to moderate malaria risk areas
Source: New Microbes New Infect. 2025 Jan 1;63:101561. doi: 10.1016/j.nmni.2024.101561 (PMC11840869; doi:10.1016/j.nmni.2024.101561)
Supplement: Multimedia component 3 [file mmc3.pdf]

Supplementary Table S3. Characteristics of travellers who experienced fever during travel to moderate-risk malaria endemic areas (n=25).

Nr	Sex	Age[a]	Country visited[b,c]	Fever start >7 days after arrival?[d,e]	Duration fever[d]	Highest temp measured	Method of fever measuring	Other symptoms	SBET[f]	Doctor visit	Treatment taken	Diagnosis	Dried bloodspot[g]	Blood Sample[h]
**Participants 1-15 advised in accordance to LCR guidelines 2017 until September 2021[i]**														
1	M	30-35y	Colombia	Yes	2 days	Not measured	N.A.	Feverish, headache, diarrhoea	N.A.	No	Ibuprofen, PCM	Sinusitis	No	Not taken
2	F	25-30y	Guatemala	Yes	2 days	38.5°C	Rectal	Headache, nausea, diarrhoea, muscle pain, fatigue	N.A.	No	No	No	No	64 days
3	F	40-45y	Thailand	Yes	1 day	38.5°C	Oral	Coughing	N.A.	No	No	No	No	64 days
4	M	25-30y	Thailand[c]	Yes	3 days	37.9°C	Oral	Feverish, headache	N.A.	No	No	No	No	Not taken
5	F	25-30y	Guatemala	Yes	1 day	38.7°C	Rectal	Headache, nausea, muscle pain, feeling week	N.A.	No	No	No	No	163 days
6	F	30-35y	Philippines	No	2 days	Not measured	N.A.	Headache, nausea, diarrhoea, muscle pain	N.A.	No	No	No	No	Not taken
7	F	30-35y	Indonesia	Yes	1 day	Not measured	N.A.	Nausea, diarrhoea	N.A.	No	No	No	No	29 days
8	M	50-55y	Thailand	Yes	1 day	38.5°C	Rectal	Nausea, vomiting, muscle pain, dizziness	N.A.	No	No	No	No	Not taken
9	M	40-45y	India	No	2 days	39.0°C	Oral	Headache, muscle pain	N.A.	Yes	Heparin	Virus infection	No	57 days
10	M	30-35y	Thailand	Yes	1 day	38.1°C	Oral	Feverish, headache, sore throat	N.A.	Yes	No	No	No	23 days
11	F	25-30y	Thailand	No	1 day	39.0°C	Oral	Headache, nausea, muscle pain	N.A.	No	No	No	No	27 days
12	M	35-40y	Vietnam[c]	Yes	3 days	39.1°C	Oral	Nausea, vomiting, diarrhoea, muscle pain, feeling week	N.A.	No	No	No	No	31 days
13	F	20-25y	Guatemala	No	1 day	38.7°C	Rectal	Feeling week, muscle pain	N.A.	No	No	No	0 days	40 days
14	F	60-65y	India[c]	Yes	1 day	38.2°C	Oral	Feverish, headache, nausea, swollen glands	N.A.	No	No	No	49 days	49 days
15	F	30-35y	Philippines	Yes	1 day	38.6°C	Under the armpit	Headache, nausea, vomiting, fatigue	N.A.	Yes	Antibiotic	Gastro-enteritis	No	Not taken

Nr	Sex	Age[a]	Country visited[b,c]	Fever start >7 days after arrival?[d,e]	Duration fever[d]	Highest temp measured	Method of fever measuring	Other symptoms	SBET[f]	Doctor visit	Treatment taken	Diagnosis	Dried bloodspot[g]	Blood Sample[h]
**Participants 16-25 advised in accordance to LCR guidelines from September 2021[i]**														
16	F	18-20y	Thailand	Yes	1 day	38.6°C	Rectal	Headache, nausea, diarrhoea, light headed	Yes	No	No	No	No	44 days
17	F	20-25y	Indonesia[c]	Yes	1 day	39.0°C	Oral	Headache, muscle pain	Yes	No	No	Covid-19	No	42 days
18	M	30-35y	Namibia[c]	Yes	1 day	38.6°C	Oral	Diarrhoea, abdominal pain	Yes	No	No	No	No	24 days
19	M	30-35y	Indonesia	Yes	2 days	38.6°C	Rectal	Headache, nausea, vomiting	Yes	No	No	No	0 days	58 days
20	M	25-30y	Guatemala	Yes	1 day	38.5°C	Oral	Feverish, headache, nausea, vomiting, diarrhoea	Yes	No	Loperamide, PCM	No	No	20 days
21	M	25-30y	Thailand	Yes	2 days	38.2°C	Oral	Feverish, nausea, vomiting, diarrhoea	No	No	No	No	0 days	13 days
22	M	25-30y	Thailand	No	2 days	38.8°C	Oral	Headache, diarrhoea	Yes	No	No	No	No	Not taken
23	F	65-70y	Indonesia[c]	Yes	1 day	39.1°C	Rectal	Headache	Yes	Yes	No	Virus infection	No	22 days
24	M	30-35y	Indonesia	Yes	1 day	38.5°C	Oral	Headache, nausea, diarrhoea, abdominal pain	No	No	No	No	No	Not taken
25	M	30-35y	Colombia	Yes	1 days	39.0°C	Under the armpit	Headache, nausea, muscle pain. Contact with insurance and local pharmacy.	Yes	Yes	No	No	No	80 days

Abbreviations: F=female, LCR= Dutch Coordination Centre for Travellers' Health Advice, M=male, N.A.=not applicable, neg=negative, Nr.=number, PCM=paracetamol, SBET= standby emergency treatment, temp=temperature, y=year.

a. Age at moment of pre-travel consultation at the Travel Clinic of the Public Health Service of Amsterdam.
b. Country where fever started.
c. If the fever began in the Netherlands, the last visited country is specified.
d. Fever defined as measured temperature ≥38.5°C (101.3°F) or feeling feverish. A thermometer was provided during pre-travel consultation.
e. This division is based on the LCR guidelines, as the minimum incubation period for malaria is 7 days. Fever within the first 7 days may not be attributed to malaria acquired during this travel.
f. The question whether SBET was carried during travel was only asked during the 2021 LCR guidelines and in case of fever. None of the participants used the SBET during fever.
g. A dried blood spot card (Whatman 903 Proteinsaver card) was provided to all participants during pre-travel consultation with instructions for self-collection in the event of a fever. The number of days between onset of fever and self-collection is indicated.
h. Travellers experiencing a fever during travel were contacted after return for a second blood collection. The number of days between onset of fever and collection of the second blood sample is indicated.

i. The Dutch Coordination Centre for Travellers' Health Advice (LCR) produces guidelines for travel doctors and nurses in the Netherlands. The guideline from 2017 until September 2021 stated that travellers should have an SBET during travelling to moderate malaria-endemic areas. The guidelines were updated in September 2021 and specify that only travellers to remote areas (where medical assistance cannot be reached <48 hours of fever onset) should have an SBET when traveling to moderate malaria-endemic areas.
