# Supplementary material for: Adherence to stand-by emergency treatment and mosquito protection measures in short-term travellers to moderate malaria risk areas
Source: New Microbes New Infect. 2025 Jan 1;63:101561. doi: 10.1016/j.nmni.2024.101561 (PMC11840869; doi:10.1016/j.nmni.2024.101561)
Supplement: Multimedia component 4 [file mmc4.pdf]

Supplementary Table S4. Determinants of adherence to mosquito net use[a] aimed at reducing malaria risk in travellers at risk of malaria (n=356); univariable Poisson regression analysis.

Characteristic	Participants with malaria risk during travel		Univariable regression[g]		
	Non-adherent to bed net use[a] N=337	*Adherent to bed net use[a] N=19*	*PR*	*95% CI*	*p-value*
**Sex**					
Female	204 (61%)	10 (53%)	REF		0.496
Male	133 (39%)	9 (47%)	1.36	(0.56-3.26)	
**Age**					
<29 year	162 (48%)	12 (63%)	REF		0.216
30 – 39 year	124 (37%)	3 (16%)	0.34	(0.10-1.19)	
≥ 40 year	51 (15%)	4 (21%)	1.05	(0.35-3.14)	
**Country of birth**					
Netherlands	301 (89%)	16 (84%)	REF		0.434
Other countries	34 (10%)	3 (16%)	1.61	(0.49-5.26)	
Unknown	2 (1%)	0 (0%)			
**Country of birth of parents**[b]					
Netherlands	254 (75%)	15 (79%)	REF		0.774
Other countries	80 (24%)	4 (21%)	0.85	(0.29-2.51)	
Unknown	3 (1%)	0 (0%)			
**Time from pre-travel consultation until departure**					
<2 week	99 (29%)	8 (42%)	REF		0.417
2 – 5 weeks	125 (37%)	7 (37%)	0.71	(0.27-1.90)	
≥ 5 weeks	113 (34%)	4 (21%)	0.46	(0.14-1.48)	
**National LCR guideline**[c]					
Guideline from 2017	211 (63%)	12 (63%)	REF		0.962
Revised guideline implemented in 2021	126 (37%)	7 (37%)	0.98	(0.39-2.43)	
**Reason for travel**					
Tourism	320 (95%)	17 (89%)	REF		0.301
Other travel reason (work, education, visiting friends and relatives)	1 (5%)	2 (11%)	2.09	(0.52-8.40)	
**Travel duration**					
<3 weeks	106 (31%)	10 (53%)	REF		0.154
3 weeks – 1 month	126 (37%)	6 (32%)	0.53	(0.20-1.41)	
1 – 3 months	105 (31%)	3 (16%)	0.32	(0.09-1.14)	
**Travel destination**[d,e]					
Asia	221 (66%)	9 (47%)	REF		0.109
Latin America	115 (34%)	10 (53%)	2.04	(0.85-4.90)	
Africa	1 (0%)	0 (0%)	[e]		
**SBET prescribed**					
No	94 (28%)	6 (32%)	REF		0.728
Yes	243 (72%)	13 (68%)	0.85	(0.33-2.17)	
**Symptoms during travel**					
No	222 (66%)	12 (63%)	REF		0.808
Yes	115 (34%)	7 (37%)	1.12	(0.45-2.77)	
**Fever during travel**[f]					
No	317 (94%)	17 (89%)	REF		0.418
Yes	20 (6%)	2 (11%)	1.79	(0.44-7.26)	

Abbreviations: 95% CI= 95% Confidence Interval, DEET=N,N-diethyl-3-methylbenzamide, LCR=The Dutch Coordination Centre for Travellers' Health Advice, PR=Prevalence Ratio, REF=reference group, SBET=standby emergency treatment.
Due to limited numbers in the 'unknown' category, unknown was not used in the analyses. Sum can be more or less than 100% due to rounding.

a. Bed net use was assessed by calculating the percentage of days a bed net was used during the night divided by the total travel days with malaria risk. Adherence was categorized as follows: <75% bed net use was classified as non-adherent, ≥75% bed net use was classified as adherent.
b. If one parent from participant is born in the Netherlands and one parent is born in another country, the country of parents is classified as 'other country'.
c. The Dutch Coordination Centre for Travellers' Health Advice (LCR) produces guidelines for travel doctors and nurses in the Netherlands. The guideline from 2017 until September 2021 stated that travellers should have an SBET when travelling to moderate malaria-endemic areas. The guidelines were updated in September 2021 and specify that only travellers to remote areas (where medical assistance cannot be reached <48 hours of fever onset) should have an SBET when travelling to moderate malaria-endemic areas [1].
d. If a participant visited multiple continents with malaria risk, the continent where the participant spend the most days was included.
e. As only one participant visited Africa, this category was excluded from the regression analysis.
f. Fever measured using a provided thermometer, or feeling feverish during travel up to 14 days after return.
g. None of the initial variables in univariable analysis showed a significant association with bed net use in the multivariable Poisson regression model.

Reference

1. Dutch Coordination Centre for Travellers' Health Advice (LCR), *E10 Malaria guideline*. 2024, LCR.
