# Supplementary material for: Adherence to stand-by emergency treatment and mosquito protection measures in short-term travellers to moderate malaria risk areas
Source: New Microbes New Infect. 2025 Jan 1;63:101561. doi: 10.1016/j.nmni.2024.101561 (PMC11840869; doi:10.1016/j.nmni.2024.101561)
Supplement: Multimedia component 5 [file mmc5.pdf]

Supplementary Table S5. Determinants of adherence to air-conditioning use[a] aimed at reducing malaria risk in travellers at risk of malaria (n=356); univariable and multivariable Poisson regression analysis.

Characteristic	Participants with malaria risk during travel		Univariable regression			Multivariable regression[h]		
	Non-adherent with airco use[a] N=227	*Adherent with airco use[a] N=129*	*PR*	*95% CI*	*p-value*	*PR*	*95% CI*	*p-value*
**Sex**								
Female	137 (60%)	77 (60%)	REF		0.902			
Male	90 (40%)	52 (40%)	1.02	(0.77-1.35)				
**Age**								
<29 year	107 (47%)	67 (52%)	REF		0.632			
30 – 39 year	85 (37%)	42 (33%)	0.86	(0.63-1.17)				
≥ 40 year	35 (15%)	20 (16%)	0.94	(0.63-1.41)				
**Country of birth**								
Netherlands	206 (91%)	111 (86%)	REF		0.077			
Other countries	19 (8%)	18 (14%)	1.39	(0.97-2.00)				
Unknown	2 (1%)	0 (0%)						
**Country of birth of parents[b,c]**								
Low/Middle-income country	21 (9%)	17 (13%)	REF		0.377			
High-income country (excl. Netherlands)	32 (14%)	14 (11%)	0.68	(0.39-1.19)				
Netherlands	172 (76%)	97 (75%)	0.81	(0.55-1.19)				
Unknown	2 (1%)	1 (1%)						
**Time from pre-travel consultation until departure**								
<2 week	62 (27%)	45 (35%)	REF		0.012	REF		
2 – 5 weeks	98 (43%)	34 (26%)	0.61	(0.42-0.88)		0.60	(0.42-0.85)	0.004
≥ 5 weeks	67 (30%)	50 (39%)	1.02	(0.75-1.38)		0.99	(0.75-1.32)	0.956
**National LCR guideline[d]**								
Guideline from 2017	144 (63%)	79 (61%)	REF		0.680			
Revised guideline implemented in 2021	83 (37%)	50 (39%)	1.06	(0.80-1.41)				
**Reason for travel**								
Tourism	218 (96%)	119 (92%)	REF		0.083			
Other travel reason (work, education, visiting friends and relatives)	9 (4%)	10 (8%)	1.49	(0.95-2.34)				
**Travel duration**								
<3 weeks	75 (33%)	41 (32%)	REF		0.758			
3 weeks – 1 month	81 (36%)	51 (40%)	1.09	(0.79-1.52)				
1 – 3 months	71 (31%)	129 (29%)	0.97	(0.68-1.39)				
**Travel destination[e,f]**								
Asia	121 (53%)	109 (85%)	REF		<0.001	REF		
Latin America	105 (46%)	20 (16%)	0.34	(0.22-0.52)		0.34	(0.22-0.51)	<0.001
Africa	1 (0%)	0 (0%)	[f]					
**SBET prescribed**								
No	65 (29%)	35 (27%)	REF		0.764			
Yes	162 (71%)	94 (73%)	1.05	(0.77-1.43)				
**Symptoms during travel**								
No	142 (63%)	92 (71%)	REF		0.104			
Yes	85 (37%)	37 (29%)	0.77	(0.56-1.05)				

**Fever during travel[g]**					
No	213 (94%)	121 (94%)	REF		0.990
Yes	14 (6%)	8 (6%)	1.00	(0.57-1.78)	

Abbreviations: 95% CI= 95% Confidence Interval, airco=air-conditioning, DEET= N,N-diethyl-3-methylbenzamide, LCR=The Dutch Coordination Centre for Travellers' Health Advice, PR=Prevalence Ratio, REF=reference group, SBET=standby emergency treatment.
Due to limited numbers in the 'unknown' category, unknown was not used in the analyses. Sum can be more or less than 100% due to rounding.

a. Air-conditioning use was assessed by calculating the percentage of days air-conditioning was used during the night divided by the total travel days with malaria risk. Adherence was categorized as follows: <75% air-conditioning use was classified as non-adherent, ≥75% use was classified as adherent.
b. Countries categorized by income levels for 2024 by the World Bank Country and Lending Groups [1].
c. Region of birth of parents was determined by the birth country of the parent from the lowest income country.
d. The Dutch Coordination Centre for Travellers' Health Advice (LCR) produces guidelines for travel doctors and nurses in the Netherlands. The guideline from 2017 until September 2021 stated that travellers should have an SBET when travelling to moderate malaria-endemic areas. The guidelines were updated in September 2021 and specify that only travellers to remote areas (where medical assistance cannot be reached <48 hours of fever onset) should have an SBET when travelling to moderate malaria-endemic areas [2].
e. If a participant visited multiple continents with malaria risk the continent with the most days visited was included.
f. As only one participant visited Africa, this category was excluded from the regression analysis.
g. Fever measured using a provided thermometer, or feeling during travel up to 14 days after return.
h. Variables included in the initial multivariable Poisson regression model: time from pre-travel consultation until departure, travel destination.

References

1. World Bank. World Bank Country and Lending Groups. Available at: https://datahelpdesk.worldbank.org/knowledgebase/articles/906519. [Accessed 15 August 2024].
2. Dutch Coordination Centre for Travellers' Health Advice (LCR). E10 Malaria guideline. LCR; 2024; https://mijn.lcr.nl/landing.
